# Projected habitat loss and persistence of *Heortia vitessoides* (Lepidoptera, Crambidae) in China Under CMIP6 Climate Models

**DOI:** 10.3897/BDJ.14.e182844

**Published:** 2026-03-23

**Authors:** Tian Liang, Songkai Liao, Xianzhi Wang, Chumin Chen, Xinjie Mao, Hui Chen, Leiyi Chen

**Affiliations:** 1 Guangdong Lingnanyuan Exploration & Design Co Ltd, Guangzhou, China Guangdong Lingnanyuan Exploration & Design Co Ltd Guangzhou China; 2 South China Agricultural University, Guangzhou, China South China Agricultural University Guangzhou China; 3 Guangdong Eco-Engineering Polytechnic, Guangzhou, China Guangdong Eco-Engineering Polytechnic Guangzhou China

**Keywords:** species distribution modelling, MaxEnt, climate change, pest management

## Abstract

*Heortia vitessoides* Moore is a destructive pest of *Aquilaria sinensis* (Lour.) Spreng, a significant economic crop. To understand the current and future geographical range change of this pest, we employed the maximum entropy model (MaxEnt) to assess the potential habitats of *H. vitessoides* in China, integrating global distribution data with environmental variables correlated with *H. vitessoides* occurrence. The findings demonstrated that the primary environmental factors affecting the distribution of *H. vitessoides* included the precipitation of the warmest quarter, annual precipitation, annual mean temperature and slope. Given the current climate, the potential distribution area of *H. vitessoides* in China is 96.31 × 10^4^ km^2^, representing approximately 10.03% of the total land area of the country. Projections under future climate scenarios indicate an overall contraction of suitable habitats, with the SSP245 scenario for 2050 suggesting the greatest contraction (15.64%) in the potential distribution area to 85.43 × 10^4^ km^2^. Despite this decline, *H. vitessoides* maintains a robust and persistent presence in core habitats of southern China, where it maintains stable distribution patterns, which may facilitate its persistence and local spread. Some regions, particularly in south-eastern Yunnan and Sichuan, may experience slight expansions, continuing to threaten the sustainability of *A. sinensis*. This work is crucial for monitoring and control of *H. vitessoides* in locations where it currently occurs and where it may become prevalent in the future, thereby contributing to the preservation of *Aquilaria sinensis* and its associated economic ecosystems.

## Introduction

*Heortia vitessoides* (Lepidoptera, Crambidae) is a typical oligophagous pest, specifically feeding on the foliage of *Aquilaria sinensis*. *A. sinensis* is a significant economic tree species and medicinal material in southern China, also serving as an economic crop that can be made into a natural fragrance (Liang et al. 2019). Notably, *A. sinensis* has garnered recognition as a nationally protected plant, classified as third-class endangered and second-class key wild plant by Chinese authorities. The caterpillar of *H. vitessoides* exhibits characteristics of eruptive outbreaks and voracious feeding behaviour, often causing complete defoliation during periods of infestation. When food becomes scarce, they may also feed on tender shoots and fruits. Continuous infestation by *H. vitessoides* can result in the death of entire Chinese agarwood plants, posing a significant constraint to the development of the *A. sinensis* industry (Qiao et al. 2018, Pan et al. 2023). In the last few years, the extensive establishment of artificial monoculture forests of *A. sinensis* has led to the expansion of the habitat range of *H. vitessoides*. Consequently, the issue of pest and disease infestation has gained prominence. There is an urgent need for scientific investigation to delineate the potential adaptive territories of this pest, facilitating the development of efficacious control strategies.

Species distribution models (SDMs) are usually used to predict potential habitat areas. Commonly used SDMs include CLIMEX, BIOCLIM, ENFA, RF, DOMAIN, MARS, GARP, MaxEnt etc. (Lehmann et al. 2002, Thuiller 2003, Phillips et al. 2006). Within the above models, MaxEnt provides a mathematical model, based on the theory of maximum entropy that simulates a possible geographic range of species based on occurrence data and climatic variables of existing species occurrence (Phillips et al. 2006, Zhao et al. 2024). Due to its benefits, including minimal sample size requirements, high computational speed, high operational economy and more precise forecasts, the MaxEnt ecological niche model has grown in popularity in recent years for estimating the possible habitat of species and for understanding how environmental factors affect species distribution (Elith et al. 2010, Booth 2022). It can be used in many fields, such as conservation biology, invasion biology and genetic geography (Li et al. 2009, Li et al. 2022, Thomson et al. 2024). The potential distribution of the *Ficus* pest *Perina nuda* on a national scale in China is predicted using MaxEnt model and the warmest quarterly precipitation, coldest monthly minimum temperature, annual precipitation and wettest monthly precipitation were identified as the most significant variables associated with the pest invasion (Mao et al. 2024). Fadda et al. (2023) used native range records of *Melanagromyza sojae* to predict its potential distribution in South America employing the optimised MaxEnt model, based on environmental data (Fadda et al. 2023).

Climatic factors significantly influence the spatial distribution patterns of organisms on Earth, as climate is closely linked to energy and water availability for most species (Barbet-Massin and Jetz 2014). Consequently, changes to climate could modify habitat characteristics. Therefore, climate change might significantly alter the spatial geographical ranges of species in coming decades. Current studies on *H. vitessoides* primarily focus on infestation investigation, comprehensive control, chemical communication substances and molecular biology (Qian et al. 2023, Ye et al. 2023). Previously, Xu et al. (2020) analysed the Coupled Model Intercomparison Project 5 (CMIP5) dataset and projected a potential northwards expansion of *H. vitessoides* in China employing MaxEnt with bioclimatic variables. However, predictions based on CMIP5 may no longer fully capture the future risks due to advancements in climate science and data availability. Species distribution modelling using MaxEnt requires both georeferenced occurrence records and environmental variables. For *H. vitessoides*, updated presence data are now available, improving upon previous datasets and potentially correcting sampling biases. More significantly, compared to CMIP5, the current CMIP6 ensemble exhibits higher Equilibrium Climate Sensitivity (ECS) and enhanced warming signals. This next-generation framework incorporates updated specifications for atmospheric concentrations and replaces Representative Concentration Pathways (RCPs) with Shared Socioeconomic Pathways (SSPs), which better account for future socioeconomic development and greenhouse gas emission trajectories (Gidden et al. 2019, Zhu et al. 2020, Jiang et al. 2022). The CMIP6 addresses the previous insufficiencies in linking SSPs with RCPs, thus enhancing the overall framework.

Combining the current bioclimatic variables with projected climate data, this study employed a fine-tuned MaxEnt model to project the potential geographic range of *H. vitessoides* across China under both present and future climate scenarios. The aim is to clarify the trends in the extent, area and suitability of *H. vitessoides* distribution in China, providing a theoretical foundation and practical guidance for managing its spread, as well as for monitoring, early warning and control efforts.

## Material and methods

**Species occurrence data**

**Figure 1.**
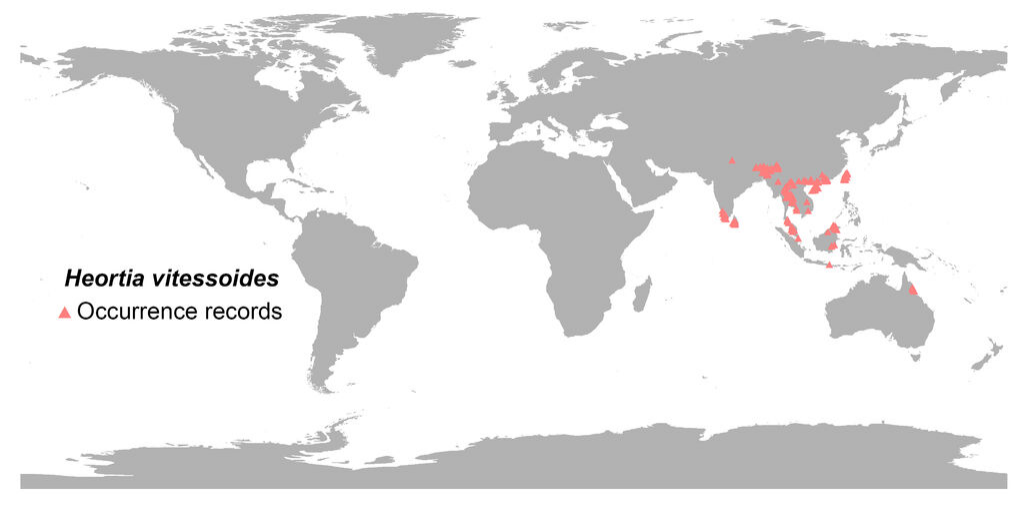
Screened geographical distribution of *Heortia vitessoides* in the world.

The occurrence data for *H. vitessoides* were obtained from two sources: (1) a review of literature on *H. vitessoides* and (2) the Global Biodiversity Information Facility (GBIF 2024). To mitigate potential issues stemming from sampling bias and spatial autocorrelation, the occurrence point data were reorganised and analysed using ArcGIS 10.8 with the help of the SDMTools plug-in. Each 10 × 10 km grid cell was limited to a single data point (Guevara et al. 2017). Ultimately, a dataset consisting of 235 verified presence location records of *H. vitessoides* were selected for analysis (Fig. 1).

**Acquisition and Selection of Bioclimate Variables**

We chose 19 bioclimatic variables (bio1-bio19), elevation dataset (elev), aspect and slope at a 5 arc-minute resolution. Slope was specifically included due to its universal ecological significance; it determines drainage patterns and solar radiation exposure, thereby shaping microclimates that constrain the spatial distribution of both insects and their host plants (Carmel and Kadmon 1999, Riihimäki et al. 2017, Wu et al. 2018). The environmental data mentioned above were sourced from WorldClim (http://www.worldclim.org/). For future projections, data for two periods were downloaded from the Beijing Climate Center's CMIP6 model: 2030s (average of 2021–2040) and 2050s (average of 2041–2060). The future climate scenarios from CMIP6 include three shared socioeconomic pathways (SSPs): SSP1-2.6 (low emissions), SSP2-4.5 (moderate) and SSP5-8.5 (high emissions). Each reflects a different combination of socioeconomic dynamics and changes in radiative forcing (Riahi et al. 2017). The topographic elevation data employed in this study was derived from the Shuttle Radar Topography Mission (SRTM) 30 m elevation data, available through the geospatial data cloud. All datasets were resampled and standardised to a uniform spatial resolution using ArcGIS 10.8. To mitigate collinearity, which can increase model complexity and reduce simulation accuracy, the 22 selected variables were subjected to Pearson correlation analysis using R version 4.2.3 (Sillero et al. 2021, Team, R.Core 2023), alongside the “ggcorrplot” v.0.1.4.1 package (Wickham 2016). Variables exhibiting correlation coefficients of |r| > 0.80 and contributing minimally to the MaxEnt model (version 3.4.1) were omitted from further analysis. Ultimately, seven key factors were retained for building the species model: Bio 18 (Precipitation of the warmest quarter), Bio 12 (Annual precipitation), Bio 1 (Annual mean temperature), Bio 2 (Mean diurnal range), Bio 3 (Isothermality), Bio 14 (Precipitation of the driest month) and slope (Table 1).

**Modelling approach**

The MaxEnt model incorporates feature combinations (FCs) and regularisation multipliers (RMs) to mitigate overfitting. Default parameters were adjusted following Peterson et al. (2018) to prevent model overparameterisation and ecological bias. Therefore, the R and the "ENMeval v.2.0" package (Kass et al. 2021) were implemented to optimise the RMs and FCs of this model, evaluate the model complexity and determine the optimal model parameters in this study (Muscarella et al. 2014). RMs were tested from 0.5 to 4.0 in increments of 0.5 and nine FCs were evaluated: L, LH, LQ, LQH, LQHP, LQHPT, LQP, QHP and QHPT. The ‘checkerboard2’ method was employed to correct the minimal Akaike Information Criterion coefficient (AICc), which measures the trade-off between model fit and complexity of this model. The optimal MaxEnt configuration (ΔAICc = 0) was selected to execute the most effective MaxEnt programme (Wei et al. 2020).

The effective occurrence points of *H. vitessoides* and the selected variables were imported to the MaxEnt model. A randomly selected 25% of the occurrence points were set aside as the testing dataset, whereas the remaining 75% data were used for training. The output file format of the model is chosen as “Cloglog”, the output format was set to “asc” and the model was replicated 10 times, while the remaining parameters were set to their default values. Using a modified Jenks' natural breaks approach in ArcGIS, the results of the MaxEnt model were divided into four classifications: unsuitable (0-0.1), poorly suitable (0.1-0.31), moderately suitable (0.31-0.6) and extremely suitable (0.6-1). The raster calculator calculates the area of each suitability level (Ji et al. 2020).

**Modelling evaluation**

Model accuracy was validated through correspondence between observed testing omission rates and predicted theoretical omission rates, with closer alignment indicating superior predictive performance (Shcheglovitova and Anderson 2013), complemented by a dual-metric assessment using the area under the curve (AUC) and the True Skill Statistic (TSS). The AUC in receiver operating characteristic (ROC) curve analysis serves as a threshold-independent measure, where the value represents the probability that a randomly chosen presence site will be ranked higher than a randomly chosen background site. Higher AUC values with the range between 0 and 1 indicated superior predictive accuracy (Ji et al. 2020). To complement AUC, we calculated the TSS, a threshold-dependent metric defined as sensitivity + specificity - 1. Unlike AUC, TSS corrects for the dependency on prevalence and provides a robust measure of prediction accuracy ranging from -1 to +1, where values nearing +1 indicate perfect agreement and values ≤ 0 indicate performance no better than random (Allouche et al. 2006).

## Results

**Model performance**

**Figure 2.**
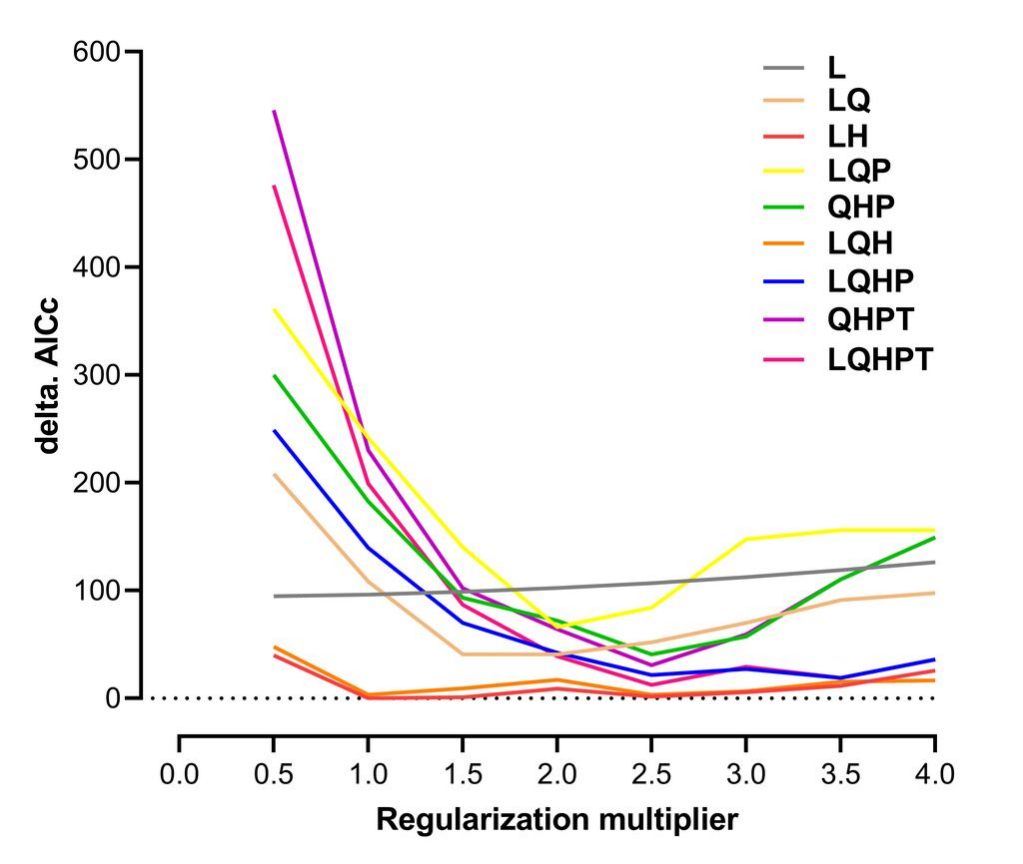
Delta Akaike Information Criterion coefficient (ΔAICc). The MaxEnt model incorporates feature combinations (FCs) and regularisation multipliers to optimise the models and prevent overfitting. In the MaxEnt model, five FCs are available for selection: linear (L), quadratic (Q), hinge (H), product (P) and threshold (T). Adjusting these parameters may significantly enhance the model's accuracy and stability. The combination of ΔAICc values = 0 was selected to run the best MaxEnt software amongst candidate models.

**Figure 3.**
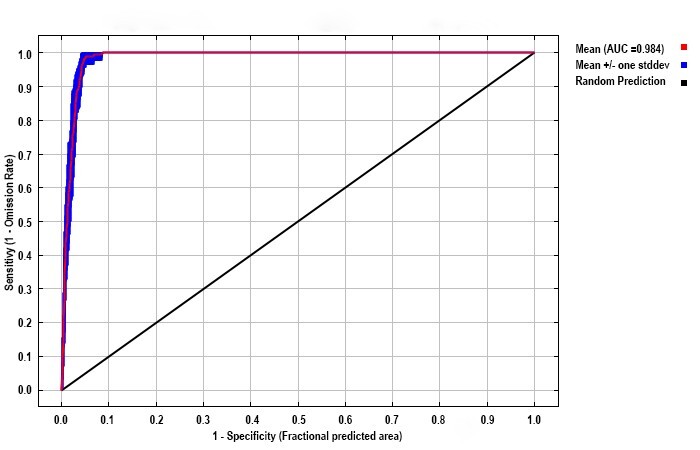
Receiver operating characteristic curves and values of the area under the curves (AUC) of the modelling. Values shown are the average over 10 replicate runs; the red line describes the average AUC of the training dataset and the blue margins show ± SD calculated over 10 replicates.

The 235 occurrence records used for modelling are spatially distributed as shown in Fig. 1. The model with RM = 1 and FC = LH had the lowest delta AICc value of 0 (Fig. 2). As shown in Fig. 3, after 10-fold cross-validation with optimised parameter settings, the mean AUC was 0.984, indicating “excellent” model predictive performance according to standard criteria (Swets 1988, Fielding and Bell 1997). These results demonstrate the excellent reliability of this model and its ability to project the potential habitat of *H. vitessoides* across China.

**Significance of the environmental variables**

**Figure 4.**
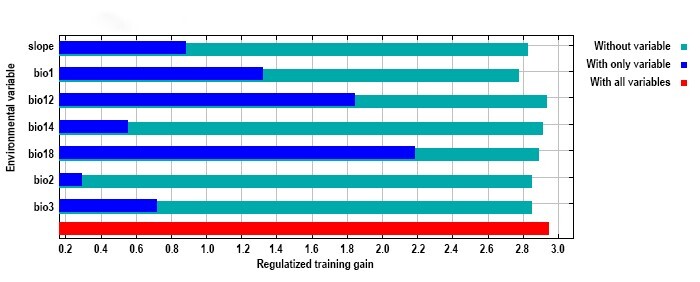
Jackknife test for evaluating the influence of environmental variables on *Heortia vitessoides* distribution prediction. Regularised training gain represents how much better the distribution fits the presence data compared with uniform distribution. ‘With only variable’ represents a result when only the particular variable is run, ‘without variable’ represents the effect of removing a particular variable from the model and ‘with all variables’ represents the results of the model when all variables are run. bio1 (Annual mean temperature), bio2 (Mean diurnal range), bio3 (Isothermality), bio10 (Mean temperature of warmest quarter), bio12 (annual precipitation), bio14 (precipitation of driest month), bio18 (Precipitation of warmest quarter) and slope.

Variable importance was assessed through: (1) relative contribution percentages and (2) jackknife tests of regularised training gain, identifying key environmental determinants of the distribution of *H. vitessoides*. The relative contributions of environmental variables are listed in Table 1 and jackknife test results of variable importance are shown in Fig. 4. Bio18 (precipitation of the warmest quarter) was the most influential variable when used alone, followed by bio12 and bio1. The omission of bio18 caused the most substantial reduction in model gain. Therefore, bio18 provided more information about the potential habitat of *H. vitessoides* than the other variables. However, the contribution of bio2 was relatively low. Combining two algorithms for evaluating variable importance, bio18, bio12, bio1 and slope were identified as the dominant environmental factors influencing *H. vitessoides* potential distribution.

**Figure 5.**
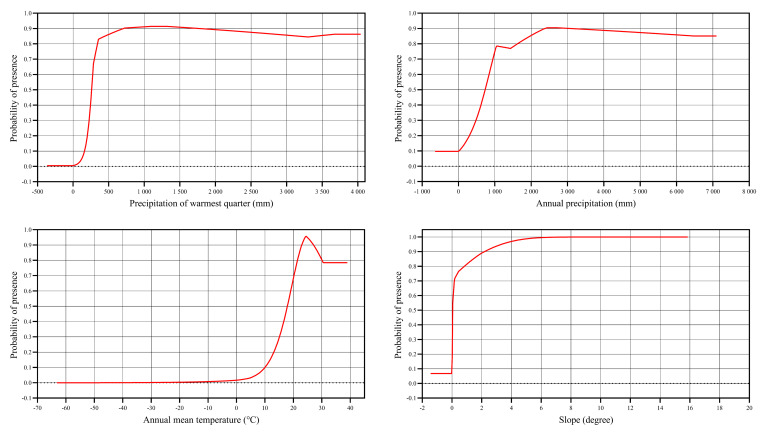
Response curves of probability of presence for *Heortia vitessoides*.

The response curve between the environmental variables (bio18, bio12, bio1 and slope) and the presence of *H. vitessoides* in the present study is shown in Fig. 5. For bio18 and bio12, the highest suitability occurred at bio18 values of 1095.73–1298.42 mm and bio12 values of 2452.14–2715.42 mm. For bio1, the highest suitability was predicted at 24.48℃. For slope, suitability values > 0.6 were predicted when the slope exceeded 0.12 degrees.

**Potential distribution of *H. vitessoides* under the current climate**

**Figure 6.**
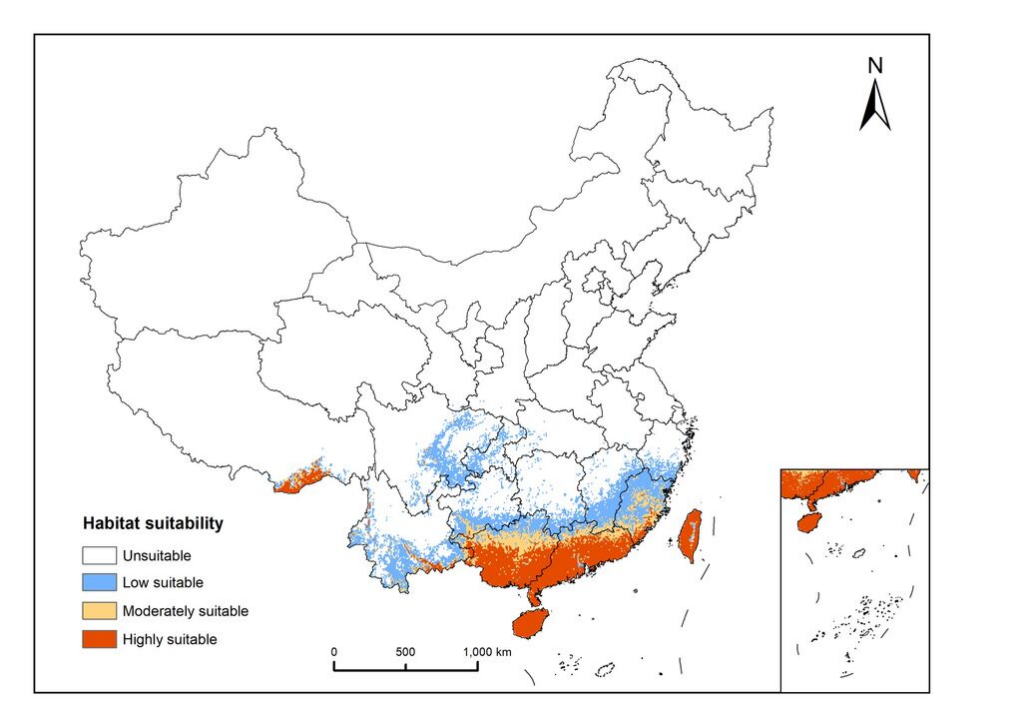
Current potential distribution of suitable habitat for *Heortia vitessoid* in China.

The results of model projections indicate that *H. vitessoides* is predominantly located in subtropical regions south of the Yangtze River (Fig. 6), amounting to approximately 9.631 × 10^5^ km^2^, representing 10.02% of China's terrestrial area. Habitat suitability is classified as follows: highly suitable (3.34%), moderately suitable (1.77%), marginally suitable (4.92%) and unsuitable (89.98%). Of these, highly suitable habitats were predominately located in Hainan, Taiwan, Guangdong, central and southern Guangxi, south-eastern border of Yunnan, south-eastern Tibet and the areas along the southern coastline of Fujian. Moderately suitable habitats were primarily distributed in central Guangxi, northern Guangdong and south-eastern Fujian. Low suitable habitats were primarily located in northern Guangxi, southern Yunnan, southern Guizhou, southern Zhejiang, eastern Sichuan, south-eastern Jiangxi, north-western Fujian and Chongqing.

**Alterations in the potential distribution of *H. vitessoides* under changing climate**

**Figure 7.**
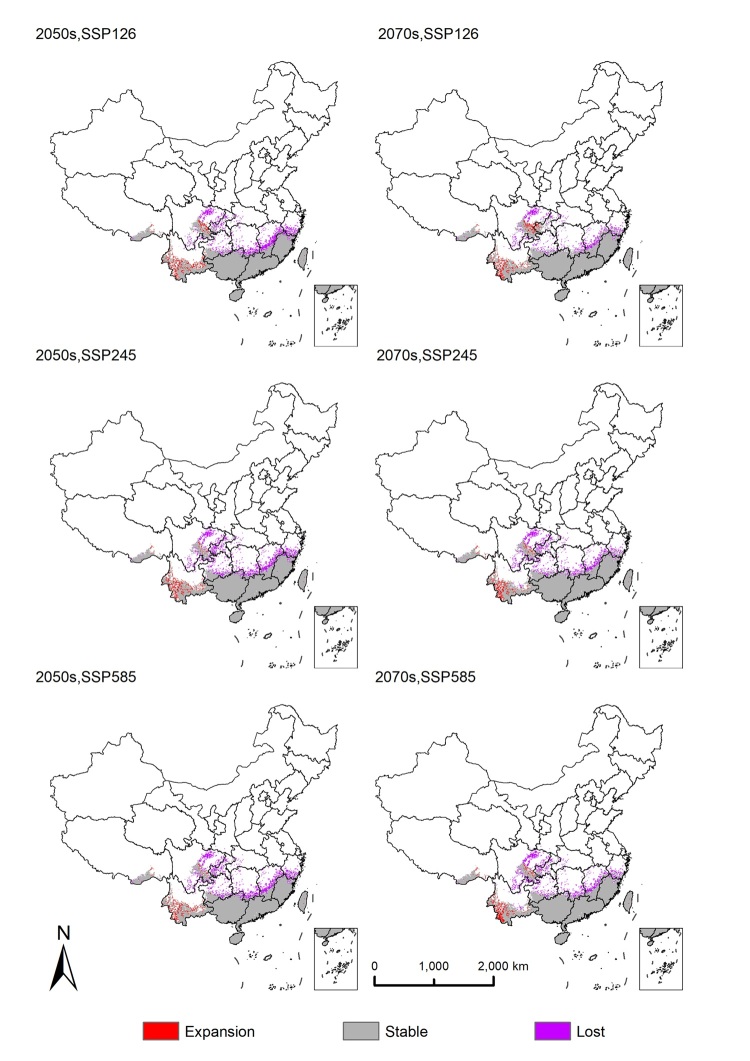
Potential suitable habitats of *Heortia vitessoid* under different climate change scenarios during the 2030s and 2050s in China.

**Figure 8.**
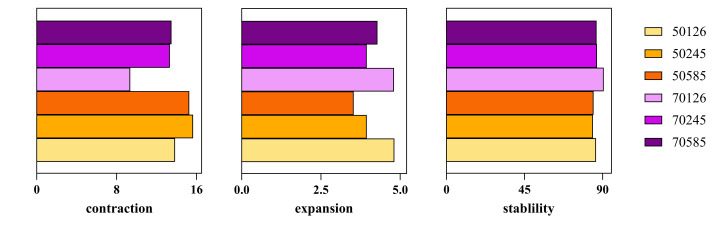
Proportion of range contraction, stability and expansion of *Heortia vitessoid* under three climate change scenarios (SSPs).

The alteration of total habitat suitability across future scenarios SSP1- 2.6, SSP2- 4.5 and SSP5- 8.5 during the 2050s and 2070s, relative to current distribution (Figs 7, 8).

The future potential suitable habitat distribution patterns for *H. vitessoides* are observed to be similar to the current distribution patterns; however, projections reveal a substantial contraction of climatically suitable habitats. Under the worst case of 2050s SSP126 scenario, *H. vitessoides* will lose about 15.64% of its current potential range. The least range losses for *H. vitessoides* are expected about 9.36% in the optimistic 2070s SSP126. In contrast, the expansion of range under all analysed scenarios does not exceed 5% relative to the current range. Furthermore, the percentages of the currently stable area remain above 85% under all future climate conditions.

In general, the habitats of expansions were primarily located in southern Yunnan and south-eastern Sichuan under future scenarios. The primary contraction habitats occurred in Chongqing, southern and eastern Sichuan, southern Guizhou, southern Hunan, southern and eastern Jiangxi and southern Zhejiang under future scenarios.

## Discussion

Understanding the potential spread of *H. vitessoides* in various locations and the suitable habitats alternatives under changing climate is crucial for surveillance, warning in advance and proactive pest management. The present study used the MaxEnt model to forecast the potential suitable habitats of *H. vitessoides* in China under current and future scenarios. The findings revealed that the model performance attained a high degree of accuracy (AUC and TSS values surpassing 0.9), demonstrating the model’s dependability and precision for projecting suitable habitats. Therefore, we believe that the performance of our model is robust enough to account for the overall distribution of suitable areas for *H. vitessoides* in China. We demonstrated that the potential suitable area for *H. vitessoides* will contract under future climate scenarios.

The MaxEnt modelling calculations revealed that the precipitation of the warmest quarter (bio18), annual precipitation (bio12), annual mean temperature (bio1) and slope were the important key environmental variables inﬂuencing the habitats of *H. vitessoides* (Table 1). Similarly, Xu et al. (2020) also indicated that bio18 is one of the crucial climatic parameters that regulate the potential spread of *H. vitessoides*. On the one hand, the precipitation and heavy rainfall directly affect insect growth, development and population dynamics (Chen et al. 2019). On the other hand, the precipitation may have an indirect impact on the environmental humidity and insect growth and spread by influencing the development of the host plants and natural predators (Park et al. 2021). In the current climate, the highly suitable habitats for *H. vitessoides* are primarily in the Pearl River Basin, which is located in the subtropics, with the Tropic of Cancer crossing the middle of the Basin, with a mild and rainy climate, average annual precipitation of 1200-2200 mm and abundant water resources (Gu et al. 2014, Lai et al. 2015). The increased precipitation and humidity favour the occurrence of *H. vitessoides*, which is compatible with the findings of this study. Furthermore, temperature is also the main factor affecting the development, survival and growth of insects (Clarke 2003). The growth and development of *H. vitessoides* are significantly influenced by temperature, within the range of 18 to 30°C, the developmental rates of the eggs, larvae, pupae and adults increasing with rising temperatures (Zhou et al. 2017). Within China's warmest quarter, adequate precipitation plays a pivotal role in fostering the growth of *A. sinensis*, thereby furnishing abundant food sources for *H. vitessoides*. Concurrently, the temperature elevation expedites the growth and maturation of *H. vitessoides*, rendering this period optimal for its survival. Slope influences drainage, soil type and sunlight exposure, thereby affecting the growth and spread of hosts and insects (Amundrud and Srivastava 2020). In our study, the occurrence probability of *H. vitessoides* rose rapidly as the slope ranged from 0 to 6 degrees, reaching a probability close to 1 at 6 degrees. This indicates its strong adaptability to varying slopes.

Xu et al. (2020) have determined that *H. vitessoides* possesses a broad range of suitable habitats within the tropical and the southern subtropical climate zone in China. The range of suitable areas identified in this study aligns closely with that reported in Xu et al. (2020) under current climate conditions. However, certain discrepancies exist between our results and their study. Specifically, Xu et al. (2020) demonstrated that the highly and moderately suitable areas of Yunnan, Guizhou and Sichuan were wider than ours and most areas of Chongqing, Hunan, Zhejiang and Hubei were separated into unsuitable habitats. In contrast, our model results classified these areas into low suitable areas.

Crucially, the findings of potential distribution under future scenarios in the current study differ from those of Xu et al. (2020). Xu et al. (2020) employed the CMIP5 database relying on the MaxEnt model to determine that the suitable distribution habitats of *H. vitessoides* would spread to northern China. In contrast, our study employed the CMIP6 database based on the MaxEnt model and predicted that the potential distribution area of *H. vitessoides* shrinks to the south as a whole. The observed differences may be attributed to the updated occurrence data of *H. vitessoides* and advancements in climate modelling. Recent evaluations indicate that CMIP6 incorporates improved physical parameterisations and new Shared Socioeconomic Pathways (SSPs), which enable more realistic simulation outcomes (Wei et al. 2022). The research findings of Jiang et al. (2020) and Xin et al. (2020) demonstrated that the CMIP6 models outperform the CMIP5 models in modelling the climate in China when comparing the climate simulation capabilities of the two models.

While CMIP5 models often projected milder warming that facilitated high-latitude range expansion, the stronger warming signals in CMIP6 likely indicate that temperatures in current southern habitats may exceed the physiological thermal tolerance of *H. vitessoides* (e.g. during the warmest quarter), shifting the projection from expansion to contraction. Consistent with climatic response curves, habitat suitability was highest at intermediate value of annual mean temperature (Bio1) and sufficient value adequate precipitation during the warmest quarter (Bio18), but decreased sharply once thermal or moisture thresholds were exceeded. Accordingly, our results show varying degrees of habitat reduction in southern and eastern Sichuan, Chongqing, Hubei, Guizhou, Hunan, Jiangxi and Zhejiang, with the most pronounced decline occurring under the SSP2-4.5 scenario in 2050. These reductions are likely attributed to the influence of global warming along with the escalated intensity and frequency of extreme weather events observed in recent years, thereby exerting an influence on the prospective distribution of *H. vitessoides* (Urban 2015).

Conversely, the predicted slight habitat expansion in south-eastern Yunnan, south-eastern Sichuan and Tibet may be attributed to the significant topographic complexity and elevational gradients characterising these regions. Complex terrain facilitates the formation of cooler microclimates and enhances precipitation via orographic uplift, enabling local environmental conditions to remain within — or shift into — the optimal ranges of key variables, such as Bio1 and Bio18, even under future warming contexts. Consequently, climate change is likely to induce spatially heterogeneous patterns of habitat response, characterised by predominant contraction in low-elevation core distributions, whereas topographical buffer zones exhibit habitat persistence or localised expansion. Given that the total suitable habitat area remains above 85% across the six climate scenarios, with certain regions retaining high suitability, it is imperative to establish continuous monitoring and early warning systems within these stable or high-risk zones. Management strategies should prioritise the long-term surveillance of stable populations and rapid responses to potential outbreak hotspots. Incorporating integrated measures, such as chemical and biological controls, is essential to mitigate the risk of localised pest outbreaks, rather than focusing solely on potential northward expansion trends (Yan et al. 2019).

Although the MaxEnt model shows excellent performance in projecting the potential distribution of *H. vitessoides*, several limitations should be acknowledged (Yan et al. 2018). Despite the application of spatial filtering to mitigate sampling bias, reliance solely on presence data may still introduce bias. Furthermore, the model primarily includes climatic and topographic variables, potentially overlooking other influencing factors such as changes in land use and human activities (Phillips et al. 2009, Sillero and Barbosa 2019, Dang et al. 2021). Additionally, our projections are based on the abiotic niche of the pest and implicitly assume a constant availability of the host plant, *Aquilaria sinensis*. However, the distribution of *A. sinensis* is also dynamic and susceptible to climate change. A potential spatial mismatch between the pest’s future climatic niche and the host’s shifting range is not accounted for in this single-species model, which might lead to an overestimation of suitable habitats in areas where the host becomes compromised. Therefore, future research needs to take these aspects into account to achieve a more comprehensive and broader understanding of the distribution dynamics of *H. vitessoides*. Additionally, there is inherent uncertainty in climate predictions, which warrants a cautious interpretation of the results. While CMIP6 models offer improved sensitivity, uncertainties remain in regional climate projections, particularly regarding precipitation patterns over the complex topography of East Asia (China). This implies that the exact delineation of the pest’s future range may be subject to local-scale uncertainties. Scenario-based modelling provides valuable insights; however, actual outcomes may differ due to unforeseen environmental changes and human activities. Long-term monitoring and adaptive management strategies will be crucial for effectively addressing changes in the distribution of *H. vitessoides*.

## Conclusions

This study employed an optimised MaxEnt model to thoroughly assess the current and future potential habitats of *H. vitessoides* in China. The findings underscore the significant roles of precipitation and temperature in shaping the pest’s distribution, predicting an overall contraction of suitable habitats under future climate scenarios. These insights hold substantial implications for developing targeted pest management strategies and protecting *A. sinensis* plantations from *H. vitessoides* infestations. Ongoing research and adaptive management are critical for alleviating the impacts of climate change on pest dynamics and ensuring the sustainability of economically important ecosystems.

## Figures and Tables

**Table 1.** Percentage contribution of each environmental variable to MaxEnt model for *Heortia vitessoides*.

Environmental variable	Percentage contribution (%)
bio 18 (Precipitation of warmest quarter)	38.9
bio 12 (Annual precipitation)	36.8
bio 1 (Annual mean temperature)	6.2
Slope	6
bio 3 (Isothermality)	5.7
bio 2 (Mean diurnal range)	4.3
bio 14 (Precipitation of driest month)	2
